# Physiochemical Characterization of Lipidic Nanoformulations Encapsulating the Antifungal Drug Natamycin

**DOI:** 10.3390/nano14080726

**Published:** 2024-04-20

**Authors:** Luigi Talarico, Ilaria Clemente, Alessandro Gennari, Giulia Gabbricci, Simone Pepi, Gemma Leone, Claudia Bonechi, Claudio Rossi, Simone Luca Mattioli, Nicola Detta, Agnese Magnani

**Affiliations:** 1 Department of Biotechnology Chemistry and Pharmacy, University of Siena, Via Aldo Moro 2, 53100 Siena, Italy; luigi.talarico@student.unisi.it (L.T.); ilaria.clemente2@unisi.it (I.C.); alessandro.gennari@student.unisi.it (A.G.); giulia.gabbricci@student.unisi.it (G.G.); pepi11@student.unisi.it (S.P.); gemma.leone@unisi.it (G.L.); claudia.bonechi@unisi.it (C.B.); claudio.rossi@unisi.it (C.R.); 2 National Interuniversity Consortium of Material Science and Technology (INSTM), Siena Research Unit, Via G. Giusti 9, 50121 Firenze, Italy; 3 Center for Colloids and Surface Science (CSGI), Siena Research Group, Via della Lastruccia 3, 50019 Sesto Fiorentino, Italy; 4 Dompé Farmaceutici S.p.A, Via Campo di Pile SNC, 67100 L’Aquila, Italy; simone.mattioli@dompe.com (S.L.M.); nicola.detta@dompe.com (N.D.)

**Keywords:** natamycin, lipid nanoparticles, encapsulation, supramolecular, NMR, SAXS

## Abstract

Natamycin is a tetraene polyene that exploits its antifungal properties by irreversibly binding components of fungal cell walls, blocking the growth of infections. However, topical ocular treatments with natamycin require frequent application due to the low ability of this molecule to permeate the ocular membrane. This limitation has limited the use of natamycin as an antimycotic drug, despite it being one of the most powerful known antimycotic agents. In this work, different lipidic nanoformulations consisting of transethosomes or lipid nanoparticles containing natamycin are proposed as carriers for optical topical administration. Size, stability and zeta potential were characterized via dynamic light scattering, the supramolecular structure was investigated via small- and wide-angle X-ray scattering and 1H-NMR, and the encapsulation efficiencies of the four proposed formulations were determined via HPLC-DAD.

## 1. Introduction

Among common causes of blindness, ocular infectious diseases constitute some of the most widespread conditions. Particularly, fungal keratitis (FK) is a severe corneal infection that leads to visual impairment and blindness, with a substantial impact on the quality of life of patients and on healthcare costs. It affects approximately one million people worldwide; the majority of cases are found in developing countries with low-rate income in Asia and Africa [1]. The most frequent causes are infections due to ocular injuries caused by organic matter (e.g., during harvesting), low hygiene conditions in the use of contact lenses [2] and immunosuppressive diseases [3,4]. The fungi responsible for the majority of infections (95%) are *Aspergillus* and *Fusarum*, but more than 393 species of fungi are related to the development of FK [5,6]. Fungal keratitis is an infection mainly spread among developing countries that leads to unilateral blindness in severe cases and has severe social consequences, as it is directly linked to a higher risk of work-related injuries and it greatly impacts healthcare costs, including in the Western world.

Antimycotic agents such as voriconazole are already used off label in topical eye drops, but fungal keratitis can also be treated with chlorhexidine and itraconazole, either administered in a systemic or topical formulation [7,8,9], even though concentration-dependent toxicity has been reported for these compounds [10]. Natamycin is a strong antimycotic agent already available in commercial formulations, being the only FDA-approved treatment of ocular fungal infections. As of today, it is considered one of the most potent antimycotic compounds, as its very low reported minimum inhibition concentration, i.e., 0.5–6 ppm, is sufficient for molds and yeasts, while its observed inhibitory concentrations for aspergillus and fusarium strains are both in the parts per million magnitude order [11,12,13]. Its antimycotic effect is exploited without altering cellular membrane permeability [14], by binding irreversibly with ergosterol and forming an ergosterol–polyene complex inhibiting the cellular membrane [15,16]. NAT affinity with ergosterol is mainly due to the presence of double bonds in the sterol B-ring; as observed by Welsher et al., this complex’s structure is not yet well defined [14,16]. Natamycin is mainly constituted by a polyene moiety, a mycosamine group and a carboxylic acid that make it an amphoteric molecule with an isoelectric point of 6.35 and very poor water solubility (40 µg/mL) [12,17]. For this reason, the approved formulation of natamycin, sold as Natacyn^®^ (natamycin ophthalmic suspension 5%) requires frequent applications (every 1–2 h) in the first and crucial stage of treatment. Like the majority of polyenes, natamycin is easily degraded by UV exposure, pH changes and temperature oscillations [18] and its degradation products are biologically inactive. Indeed, its effectiveness as an ophthalmic drug is often hindered both by its physicochemical properties, e.g., low bioavailability and low retention at the ocular surface, and by the peculiar anatomy of the human eye, whose complexity constitutes a major challenge in drug delivery [19]. These limitations affect the clinical utility of natamycin despite its potent antifungal activity, and even though several administration routes have been used to improve the permeability of molecules across ocular anatomical and physiological barriers, traditionally popular techniques, e.g., systemic or topical administration and injections often involve complications [20]. Thus, the use of nanocarriers employing various formulation strategies has been proposed, to improve the pharmacokinetic profile and enhance the targeted delivery and drug safety. Indeed, nanoparticles can reduce the administration frequency, perform a controlled drug release and improve cellular uptake and bioavailability of compounds. Particularly, lipid-based nanoparticles possess good membrane permeation abilities, high biocompatibility and drug-loading content and low cytotoxicity [21]. Several types of lipid nanosystems with different supramolecular aggregation, surface charge and composition have shown to be promising candidates as vectors both for the anterior and posterior segment of the eye [22].

Conventional liposomes made of a single type of lipid building block have shown low corneal permeation capability, and various efforts have been made to enhance their carrier efficiency. To prevent the accumulation of carriers on the skin surface, edge activators such as SPAN, TWEEN surfactants or other membrane components such as cholesterol and sodium cholate are usually employed in nanovesicle design. Liposomes with an edge activator are called transferosomes [23], and when ethanol is present in the formulation, those nanosystems are called transethosomes (TEs). The presence of ethanol in the lipid bilayer helps with the fluidification of the system, and it is already approved for ophthalmic administration, as it is used on the eye surface in various kinds of surgery [24,25]. Transethosomes formulations for drug delivery have already found applications with several bioactives [26,27,28] and showed high permeation rates through the skin [29]. Lipid nanoparticles (LNPs) formulated with cationic ionizable lipids and helper components are particularly efficient vectors for a wide range of compounds, due to their peculiarity to maintain a neutral charge at physiological pH, possessing pKa < 7 but become charged at even slightly acidic pH found in inflammatory tissues, thus showing low cytotoxicity [30]. Moreover, this property facilitates adhesion and retention to negatively charged mucus layers such as the ocular surface, thus increasing prolonged release and uptake. In this work, two types of lipid-based formulations, i.e., transethosomes and LNPs were synthesized and characterized, and their properties were evaluated as carriers for natamycin [30,31].

## 2. Materials and Methods

### 2.1. Materials

Natamycin (NAT) (Pharmaceutical Secondary Standard, CRM), soy PC (95%. Avanti Polar Lipids, Alabaster, AL, USA), 1,2-distearoyl-sn-glycero-3-phosphocholine (DSPC) (>99% Avanti Polar Lipids, Alabaster, AL, USA), 1,2-dioleyloxy-3-dimethylaminopropane (DODMA) (>99% Avanti Polar Lipids, Alabaster, AL, USA), cholesterol (>99% Avanti Polar Lipids, Alabaster, AL, USA), sodium cholate (from bovine and/or ovine bile, 99%), ethanol absolute (for analysis), methanol (gradient grade), acetonitrile (LC-MS grade), formic Acid (98–100% for HPLC LiChropur™ Sigma-Aldrich, Milano, Italy) and MWCO membranes (14 kDa) were all purchased from Sigma-Aldrich (Milano, Italy). Phree—phospholipid removal cartridges were acquired from Phenomenex (Torrance, CA, USA).

### 2.2. Methods

#### 2.2.1. Synthesis of Natamycin-Loaded Transethosomes

An ethanolic solution of Soy PC (435 mg in 1.3 mL) was mixed with the aqueous phase of sodium cholate (0.104 mg in 3 mL). The mixture was kept under magnetic stirring for 1 h at 37 °C in a thermostatic bath to create a pre-emulsion. The resulting suspension was sonicated with a Bandelin Sonoplus UW2070 at 40% power, for 5 min and cycle intervals of 60%. Empty transethosomal samples were abbreviated as TE. Natamycin-loaded transethosmes (TE-NAT) were prepared adding 500 µL of natamycin solution (1 mg/mL) in ethanol acidified with formic acid 0.1% *v*/*v* before sonication. Samples were then freeze dried and resuspended in 4 mL H_2_O and extruded with a 100 nm polycarbonate membrane with LiposoFast apparatus.

#### 2.2.2. Synthesis of Natamycin-Loaded Lipid Nanoparticles

Three different molar ratios of lipid components were tested to evaluate the influence of each component on structural organization, cargo loading and encapsulation efficiency. The chosen molar ratios were 9:6:5, 9:5:6 and 10:4:6 of cholesterol, DODMA and DSPC, respectively, dissolved in ethanol to obtain a total lipid concentration of 20 mM for all samples (namely LNP1, LNP2 and LNP3). In loaded samples 1 × 10^−3^ M of natamycin (1 mg/mL in ethanol stock solution) was added, and the solvent was then evaporated to obtain a dry lipid film. The samples were resuspended in citrate buffer (pH 5.5), vortexed and homogenized firstly with 5 freeze-and-thaw cycles and then sonicated for 3 cycles of 3 min each with a Bandelin Sonoplus UW2070 at 70% power, obtaining formulation LNP1-NAT, LNP2-NAT and LNP3-NAT. Table 1 resumes the composition of all formulations.

#### 2.2.3. Size and Zeta Potential Measurements

Dynamic light scattering was used to determine the hydrodynamic diameter and zeta potential of empty and natamycin-loaded transethosomes and LNPs. Measurements were performed with a Malvern Zetasizer Pro Red Label (Malvern, Worcestershire, UK), with backscatter collection angle (173°) and keeping the temperature at 25 °C. The reported values for size and zeta potential are the means of three experiments performed on each sample.

#### 2.2.4. NMR Analysis

NMR analyses were performed on a Bruker DRX-600 Avance which operates at 600.3 MHz (1H), equipped with gradient unit XYZ. The obtained data were elaborated with Bruker Topspin ver. 3.6.1. To acquire spectra of the samples, freeze-dried transethosomes were resuspended in D_2_O, while LNPs were directly synthesized in deuterated water. Reference spectra for natamycin and sodium cholate were obtained in deuterated-DMSO solutions.

#### 2.2.5. SAXS/WAXS

Small and wide-angle X-ray scattering were performed on a Xeuss 3.0 HR apparatus (Xenocs, Grenoble, France) equipped with a movable EIGER2R (1 M model) hybrid pixel photon counting detector (Dectris Ltd., Baden, Switzerland) and a Genix3D (Cu) X-ray source. Three sample-to-detector distances, i.e., 60, 300 and 1800 mm were measured to cover a continuous q-range from 0.004 to 3.26 Å^−1^, calibrating the sample to detector distance by using the well-known lamellar scattering pattern of silver behenate (d = 58.376 Å). The scattering vector q is defined as q = 4π/λ sin θ, where 2θ is the scattering angle. The average acquisition time per sample was 600 to 2100 s, and all samples and pure water were measured in sealed borosilicate glass capillaries of internal diameter of 1.5 mm and under vacuum. XSACT (X-ray scattering analysis and calculation tool) software (Xenocs, France) was used to perform data reduction, normalization, subtraction and merging. GAP (Global Analysis Program, written and made available by Prof. Georg Pabst) software was used to perform data analysis and fitting [32,33] of LNPs by using the modified Caillé theory (MCT) [34,35], that accounts for the bilayer bending rigidity, combined with a Gaussian electron density representation of the bilayer headgroups and tails (MCG). The scattering intensity is described by the following equation (Equation (1)):

(1) $$I\left(q\right)=\frac{\left(1-N_{diff}\right)S\left(q\right)P\left(q\right)+N_{diff}P(q)}{q^{2}}$$

where N_diff_ is the fraction number of uncorrelated bilayers per scattering domain, *S*(*q*) is the structure factor and *P*(*q*) is the form factor, to account for the presence of bilamellar structures combined to the diffuse scattering of unilamellar vesicles. To obtain the electron density profile of the bilayer three Gaussians are used, centered at ±zH for the position of the two polar headgroups, and at zH = 0 Å for the position of the terminal methyl groups of the hydrophobic chain, with widths of σH and σC, respectively, and ρC as the negative amplitude of the methyl terminus.

#### 2.2.6. Determination of the Encapsulation Efficiency

The encapsulation efficiencies of all the nanosystems were evaluated after purification of samples via dialysis. Purified samples were treated for the elimination of phospholipid using Phree solid phase extraction cartridges: 100 µL of transethosomal or LNPs suspension were diluted with methanol up to a total volume of 1 mL (10% (*v*/*v*) and eluted in a cartridge previously conditioned with 1 mL methanol. The resulting solution was analyzed by HPLC-DAD (Thermo Fisher UltiMate 3000, Monza, Italy) equipped with a Kinetex C18 Polar column (250 × 2.1 mm, 100 Å, 2.6 µm, Phenomenex, Torrance, CA, USA). Throughout all experiments, the column oven was maintained at a constant temperature of 40 °C. An isocratic method was employed, with eluent A consisting of water with 0.1% *v*/*v* formic acid and eluent B composed of acetonitrile with 0.1% *v*/*v* formic acid, in a 70:30 ratio. A linear calibration curve was obtained with this method between 0.1 and 10 ppm.

#### 2.2.7. In Vitro Release

A known amount of purified nanosystems were put in a dialysis bag (MWCO 14 kDa) and kept under gently stirring for 12 hours at 37 °C. The selected release medium consisted of a phosphate buffer (pH 7.4) and ethanol mixture. As reported in the literature [36], adding ethanol (20% *v*/*v*) in the dissolution medium improves solubility of lipophilic compounds. Aliquots of the dissolution medium were withdrawn at regular intervals and fresh releasing medium was added each time to maintain sink conditions. The aliquots were analyzed for the quantification of natamycin using the HPLC method described above.

## 3. Results and Discussion

### 3.1. Size, Zeta Potential and SAXS/WAXS

Results obtained from size and zeta potential determinations via dynamic light scattering are reported in Table 2. All the proposed nanosystems showed monodisperse distributions of dimensions and appropriate size suitable for topical application. The zeta potential analysis evidenced very high stability of the formulations, granted by their charge distribution.

The main observable effects of natamycin inclusion in the proposed lipidic nanoformulations are a reduction in dimension and an alteration in surface charge. Since natamycin is a zwitterionic and amphiphilic molecule, its contribution to surface charge and dimension can only be explained as an interaction with the lipidic bilayer, and a surface charge rearrangement that exposes charged groups towards the outer layer of nanoaggregates. Loaded TEs showed a smaller size with respect to the unloaded samples, while the zeta potential increased, evidencing how natamycin is in a negatively charged status and it contributes to the final properties of the formulation charge and dimensions. Both samples can be considered monodisperse, as they have a polidispersity index below 0.3, and electrostatically stable, as their zeta potential values are greater than −25 mV. The three series of LNPs showed monodisperse populations (ranging between 0.05 and 0.22) and stability up to several weeks as well, exhibiting strong positive surface charge for both unloaded and loaded systems. As has already been seen for transethosomes it can also be noticed that, for LNPs, loaded nanosystems possessed smaller size distributions with respect to empty ones (Table 2), whereas zeta potential values increased. Comparing TE and LNP formulations, the effect of NAT encapsulation shows the same trends, with a general decreasing of dimension [37] and increase in zeta potential. The main reason for this behavior is the different synthetical conditions for the two nanosystems; while TEs are dispersed in a neutral water solution, LNPs are synthetized in a citrate buffer. At these two different pH conditions, natamycin is present in two different forms, as its carboxylic acid has a pKa of 3.5 and its amino group of 9, with an isoelectric point at 6.3 pH value. The strong interaction of natamycin with the lipid matrix is evidenced by the tighter supramolecular packing induced upon encapsulation in both nanoaggregate types, thus resulting in smaller dimensions of loaded samples SAXS/WAXS.

**Figure 1:**
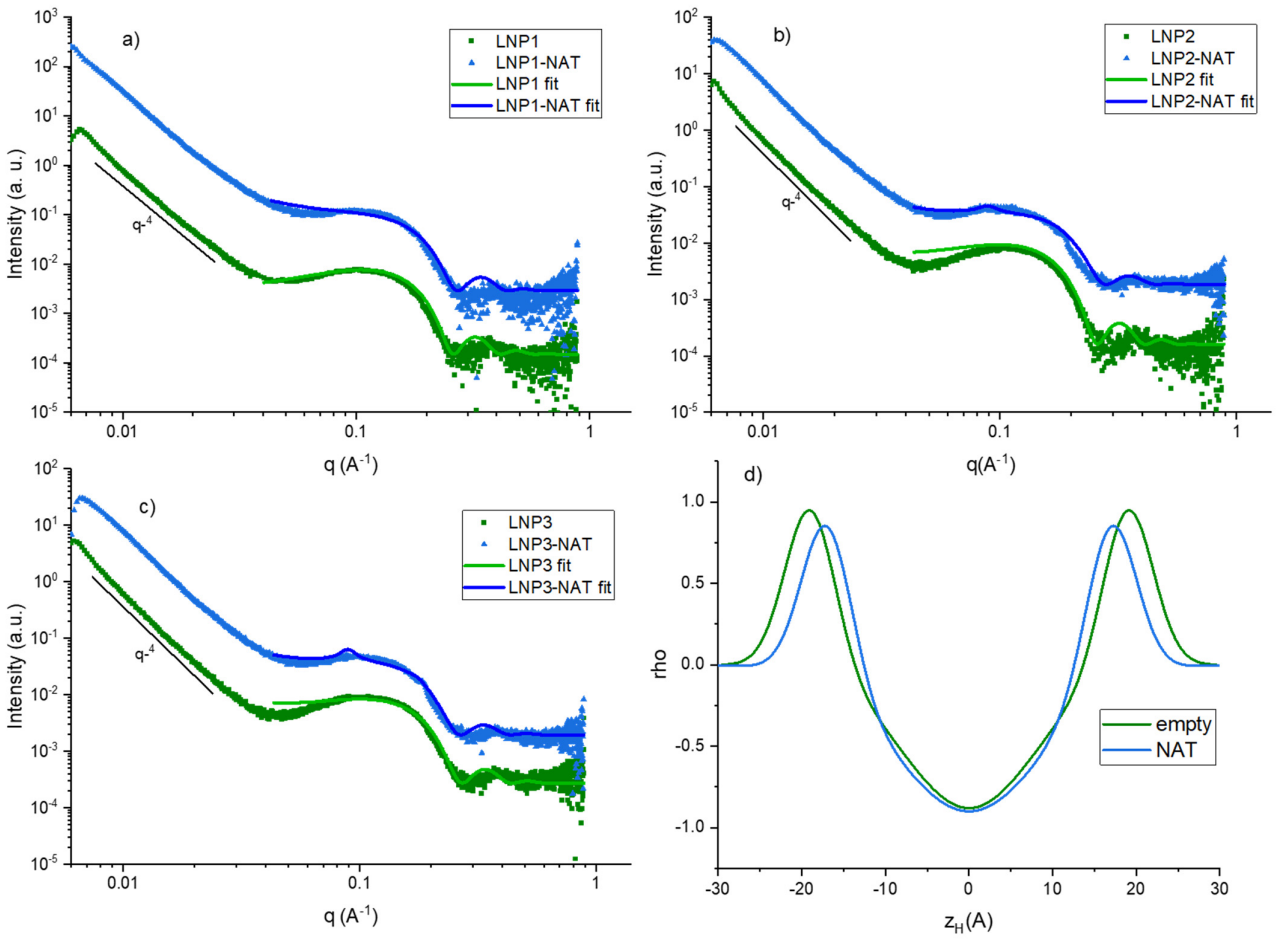
One-dimensional SAXS intensity profiles for LNP series (**a**–**c**). bilayer electron density profiles for two representative empty and NAT-loaded LNP (**d**).

SAXS intensity plots of LNPs series reported in Figure 1a–c show a typical diffuse form factor of unilamellar vesicles for all three empty samples (green curves) On the contrary, Nat-loaded samples showed small quasi-Bragg peaks from bilamellar structures superimposed to the diffuse scattering of unilamellar vesicles. This enhanced ordering became more evident going from LNP1 to LNP3, likely due to slightly higher encapsulated cargo. Indeed, the presence of loaded Nat interacting with the membrane induced bilayer structuring and enhanced bending rigidity, by intercalating in the lipid matrix and closely packing with its constituents. This was evidenced also by the electron density profiles shown in Figure 1d, where the bilayer of a representative empty sample is compared to the bilayer of its corresponding Nat-loaded sample. The reported plots showed that the maxima of the Gaussians corresponding to the polar headgroups in Nat-LNPs are shifted at smaller distances (zH) from the center of the bilayer, where the terminal methyl groups of the hydrophobic chains are located, thus indicating tighter packing. At the same time, the Gaussians corresponding to electron density of the polar headgroups showed the same widths in the two samples, evidencing that the packing only involves the hydrocarbon tails. This result suggested that natamycin preferentially intercalates the lipid bilayer by interaction mediated by its hydrophobic moiety. Tighter packing is in accordance with DLS data, that evidenced a size decrease at the supramolecular level with cargo loading. On the other hand, the rearrangement of surface charges shown by the zeta potential measurements was not dramatic at the bilayer level, indicating that the redistribution mostly influenced the charge at supramolecular level. This is likely due to the presence of associated counterions on the surface of the lipid nanosystems, stabilizing the dispersed systems with the encapsulated molecule. Regarding the obtained fitting parameters, the bilayer thickness was 3.5–4 nm for all samples, whereas the mean lamellar spacing (repeating distance) for bilamellar samples was 70 nm. These values are in accordance with supramolecular parameters obtained by light scattering. In the intermediate q region, all samples displayed a q^−4^ slope typical of sharp or smooth interfaces; this is expected for vesicular systems at these length scales [32,33].

**Figure 2:**
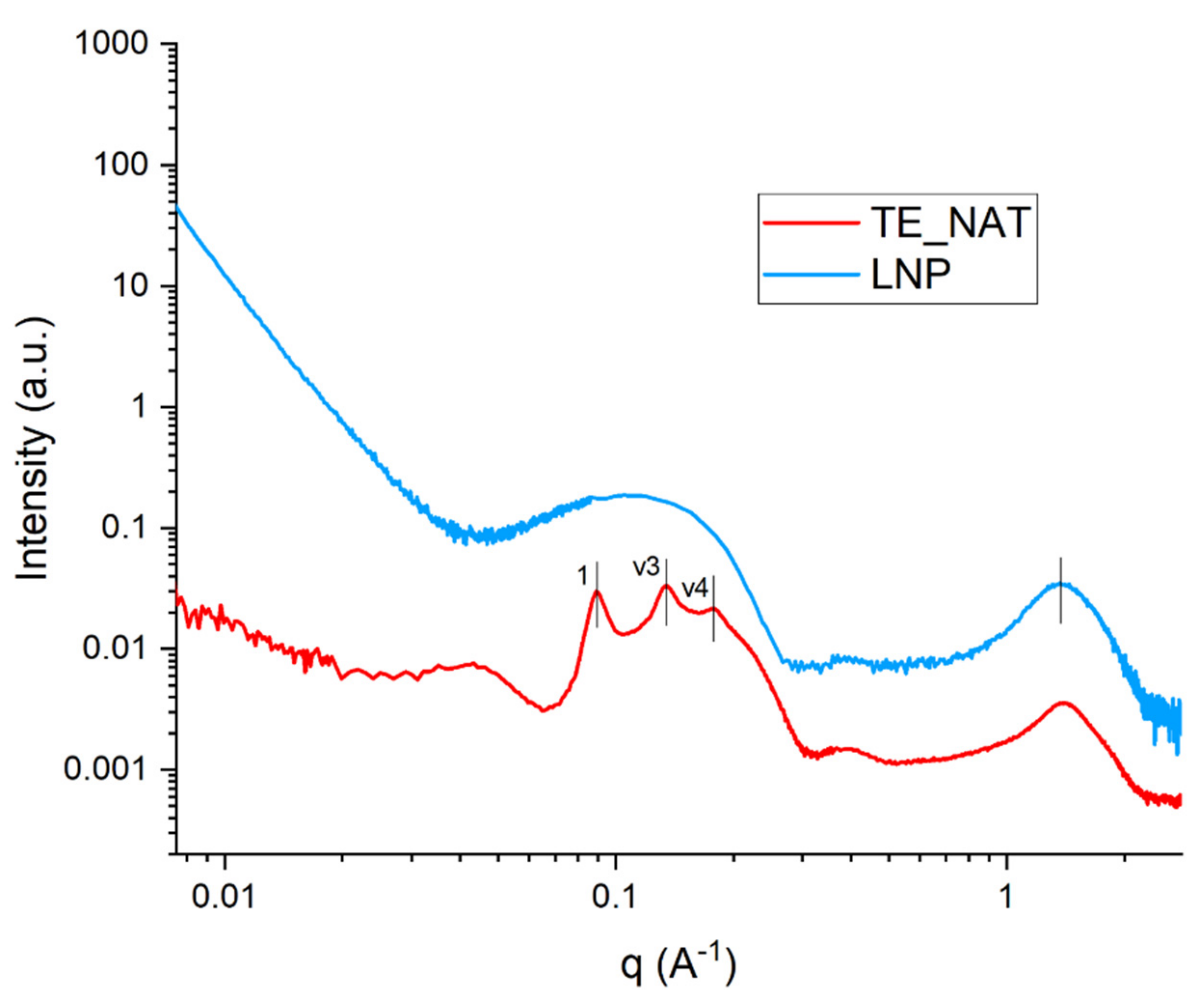
One-dimensional SAXS/WAXS intensity profiles for TEs and LNPs.

TE showed a nonlamellar arrangement superimposed to a diffuse scattering background (Figure 2, red curve), evidenced by Bragg peaks with spacing ratio $1:\sqrt{3}:\sqrt{4}$ attributable to hexagonal phases [38], thus evidencing the presence of hexagonally arranged structures (hexosomes). This arrangement is constituted by packed bilayered cylinders disposed in a hexagonal lattice, with one cylinder in the middle. Moreover, in the WAXS range at high q values (around 1.40 Å^−1^) reported in Figure 2 it is possible to notice a broad bump in both representative TE and LNP samples, that was seen for all samples, and it is typical of the packing of lipid chains at the bilayer level in soft matter aggregates, with an average spacing of about 4.45 Å.

### 3.2. NMR Analysis

**Figure 3:**
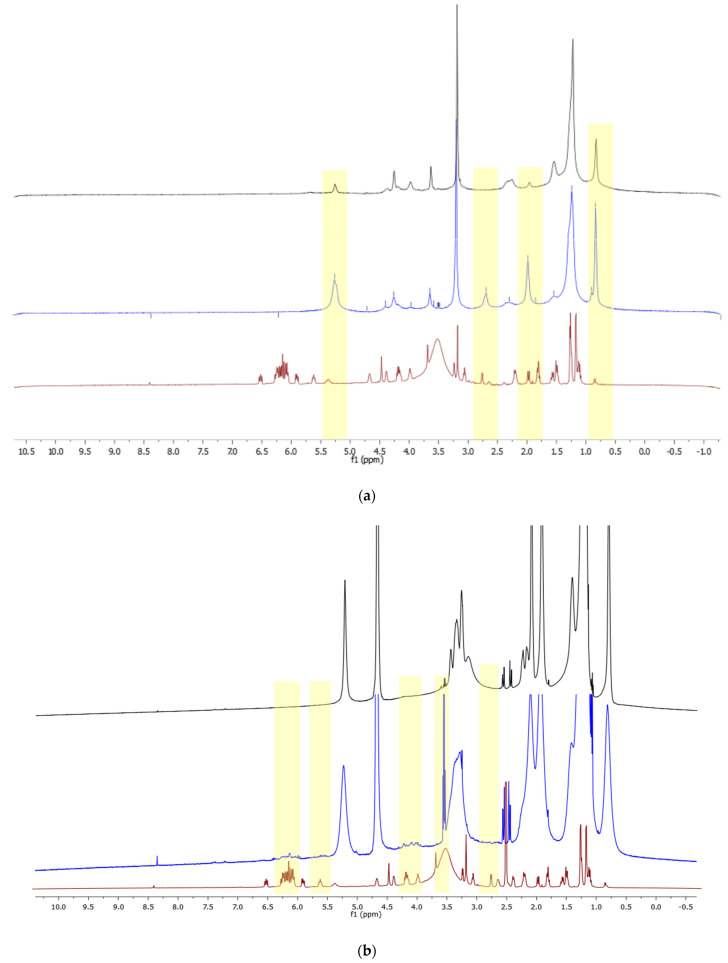
H NMR spectra of natamycin (red) TE-NAT (blue) and empty TE (black) (**a**), 1H NMR spectra of natamycin (red) LNP2-Nat (blue) and LNP2 (black) (**b**). Yellow highlighted regions are evidencing the influence of natamycin on nanoparticles spectra.

To study the conformational and dynamic properties of empty and loaded nanosystems, and the interactions of natamycin with the lipid membrane monodimensional and NOESY proton NMR spectra were recorded at 600 MHz and 298 K. As reported in the literature for dispersed nanosystems, line broadening occurred for both TE and LNP systems due to slow relaxation times. Indeed, considering the supramolecular arrangement and dimensions of the two nanocarrier types, the averaging of the anisotropic interactions was not expected to be so efficient as to avoid broadening of signals [39]. However, it was possible to perform assignment of most relevant peaks, and to extract information of natamycin localization and interactions with the bilayer. In Figure 3 the superimposed spectra of natamycin and loaded and unloaded transethosomes is reported. Peak assignment of natamycin was performed as previously reported in the literature [40,41,42,43], and each proton was named as reported in Figure S1 in the Supplementary Materials.

The superimposition of proton spectra for TE-NAT in Figure 3a evidence how signals attributable to the hydrophilic moieties of natamycin (H1′–H6′, H14, H15) have a more important influence on the natamycin-loaded transethosomes spectra, while signals corresponding to the lipophilic part of the molecule (protons from H16-H23, between 6–7 ppm) are not visible. Contrarily, in Figure 3b the spectrum of LNP-Nat showed the presence of signals (H16–H23) in the 5.5–6.5 ppm range attributable to the lipophilic moieties of natamycin, suggesting the intercalation in the LNP bilayer. Moreover, the signals at around 3–4 ppm (H2′, H11, H13) and around 2.5–3 ppm (H5, H3′) are visible as well in the LNP-Nat spectrum. The broadening of signals corresponding to the lipid alkyl chains in the loaded sample with respect to the empty one confirmed the tighter molecular packing already evidenced by DLS and SAXS data, thus resulting in slower dynamic motions of the lipid bilayer in loaded systems [44,45]. 1H NMR spectra evidenced that natamycin is able to intercalate the bilayer in both transethosomes and LNP systems, but its interactions and displayed signals depend on the system composition and aggregation.

As similarly reported for other drug-carrier systems [46], the hypothesis of natamycin being intercalated in the TE-Nat lipid double-layer membrane was further confirmed via NOESY experiments (Figure S2 in the Supplementary Materials), which suggested the spatial proximity of hydrophilic moieties of natamycin and the polar head of phospholipids or the second membrane agent (sodium cholate).

Regarding LNP samples, a representative NOESY spectrum is reported in the Supplementary Materials, Figure S3. The NMR measurements evidenced that the signals corresponding to the protons of the lipophilic moiety of Nat (H17–H22, 6.1–6.5 ppm) possessed the same sign, thus the same rate of molecular tumbling of the lipid nanosystem, coupling with the signals of lipid alkyl chains in the range 5.5–5.7 ppm (H10 DODMA/DSPC). This represents typical evidence of association of a small molecule with a macromolecule or a supramolecular aggregate [39] that further corroborates the intercalation of natamycin in the lipid bilayer, as also observed by Ciesielski et al. using DOPC/Cholesterol liposomes [47] and the absence of free drug in solution. 

Using the described purification method, the overall entrapment efficiency of the proposed systems for natamycin was quantified as 12 ± 2% for transethosomes and 81 ± 1% for LNPs.

**Figure 4:**
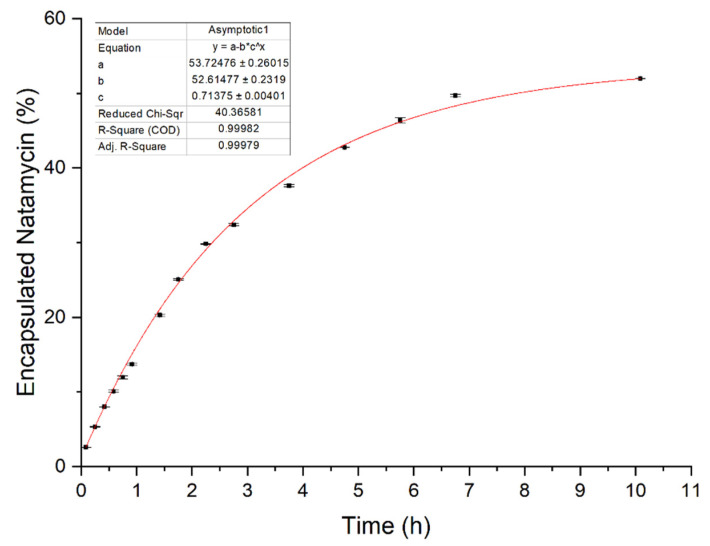
Natamycin release profile from transethosomes, obtained using a release medium composed of 20% *v*/*v* ethanol and phosphate buffer pH 7.4.

Purified transethosomes samples were used for the determination of the release profile shown in Figure 4 where the released natamycin is reported as a function of the overall encapsulated molecule. Fitted data of natamycin release from the transethosomal formulation converged for an asymptotic exponential function, as shown in Equation (2) (R^2^ = 0.999):

(2) $$y=53.7-52.6\times 0.7^{x}$$

The release was not reported for LNPs since very low release values were obtained using 20% *v*/*v* ethanol release medium, thus it was decided to not increase ethanol concentration as to avoid complete disassembly of the systems and model conditions too different from the chosen application. Indeed, LNPs showed quite high resistance and stability in these conditions.

## 4. Conclusions

In this study, two types of lipid aggregates with different supramolecular organization, namely transethosomes and lipid nanoparticles, were prepared and characterized, and their suitability as carriers for the antifungal drug natamycin was preliminarily evaluated. Physicochemical characterizations evidenced that both types of lipid nanosystem possessed interesting properties as vectors for eye treatment via topical administration. Indeed, TEs and LNPs showed appropriate dimensions (100–200 nm), monodispersity and stability in suspension as a result of their high zeta potential values and appropriate charges to facilitate the interaction either with the anterior or posterior ocular segments. Moreover, DLS and zeta potential experiments gave insights on the influence of natamycin encapsulation on size and charge distribution. SAXS/WAXS analysis revealed the different supramolecular structure of the two nanocarrier types and confirmed that the intercalation of natamycin in the bilayer can induce tighter packing and enhance structuring properties. 1H NMR evidenced the presence of signals associated to both hydrophilic and lipophilic moieties of natamycin in loaded transethosomes and LNPs, showing that the antifungal was indeed intercalated in the bilayer; this was further confirmed by NOESY measurements. Finally, the encapsulation efficiency and release properties were evaluated; both nanosystems evidenced good encapsulation capability and the TEs displayed slow and sustained release properties. In conclusion, this work has showed that transethosomes and lipid nanoparticles are appropriate candidates as drug delivery vectors for the antifungal drug natamycin, representing a valid alternative to conventional treatments for topical administration to the human eye.

## Figures and Tables

**Table 1:** Samples names and compositions.

Sample Name	Helper Lipid	Membrane Lipid 1	Membrane Lipid 2	Composition (Helper:Lipid1:Lipid2)	NAT (mg)
TE	Sodium Cholate	Soy PC	-	1:4	-
TE-NAT	Sodium Cholate	Soy PC	-	1:4	0.5
LNP1	Cholesterol	DODMA	DSPC	9:6:5	-
LNP2	Cholesterol	DODMA	DSPC	9:5:6	-
LNP3	Cholesterol	DODMA	DSPC	10:4:6	-
LNP1-NAT	Cholesterol	DODMA	DSPC	9:6:5	0.7
LNP2-NAT	Cholesterol	DODMA	DSPC	9:5:6	0.7
LNP3-NAT	Cholesterol	DODMA	DSPC	10:4:6	0.7

**Table 2:** Size, polydispersity index (PDI) and zeta potential values with respective standard deviations for natamycin-loaded and empty nanosystems.

Sample	Size (nm) ± St. Dev.	PDI ± St. Dev.	Zeta Potential (mV) ± St. Dev.
TE	158.9 ± 1.3	0.16 ± 0.074	−28.5 ± 0.6
TE-NAT	117.6 ± 1.6	0.25 ± 0.008	−35.5 ± 1.4
LNP1	212.5 ± 3	0.06 ± 0.05	+34.7 ± 1
LNP1-NAT	155.8 ± 0.7	0.14 ± 0.02	+62 ± 3.3
LNP2	259.8 ± 1.7	0.15 ± 0.01	+35.8 ± 1.2
LNP2-NAT	179.2 ± 1.7	0.22 ± 0.02	+55.2 ± 0.25
LNP3	252.2 ± 2.4	0.05 ± 0.04	+26.5 ± 0.3
LNP3-NAT	146.5 ± 1.3	0.14 ± 0.008	+58 ± 1

## Data Availability

Data are contained within the article and supplementary materials.

## Supplementary Materials

The following supporting information can be downloaded at: https://www.mdpi.com/article/10.3390/nano14080726/s1, Figure S1: represents chemical structure and proton assignment for each compound used in this work; Figure S2: represents NOESY spectra of transethosomes (a) and natamycin-loaded transethosomes (b); Figure S3: represents NOESY spectra of representative NAT-loaded LNP (right) with enlargement (left) of aromatic signals range.
