# Supplementary material for: Physiochemical Characterization of Lipidic Nanoformulations Encapsulating the Antifungal Drug Natamycin
Source: Nanomaterials (Basel). 2024 Apr 20;14(8):726. doi: 10.3390/nano14080726 (PMC11053702; doi:10.3390/nano14080726)
Supplement: Supplementary file 1 [file nanomaterials-14-00726-s001.zip › nanomaterials-2937366-supplementary.pdf]

supporting material

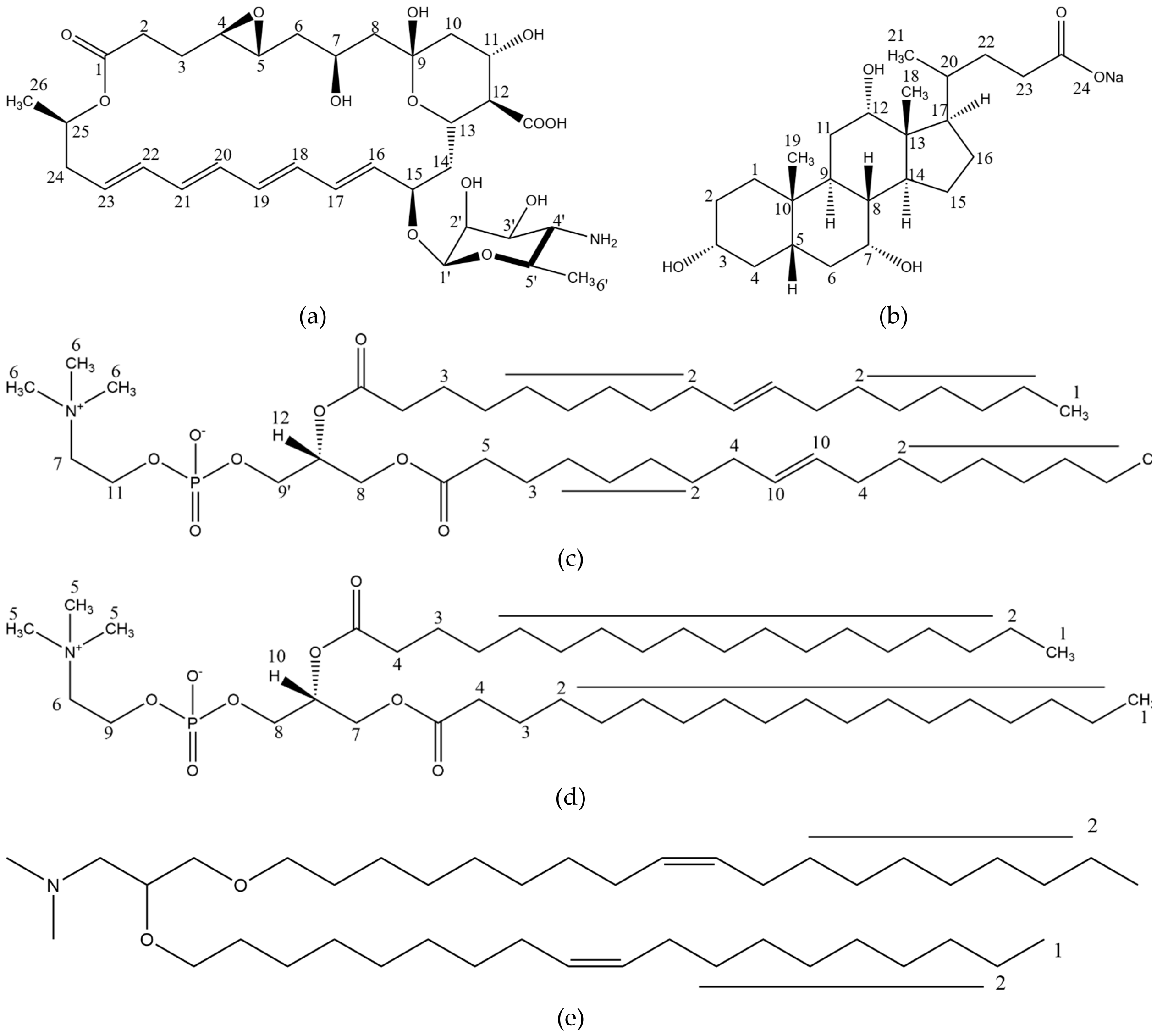

Figure S1: Structures of Natamycin (a), Sodium Cholate (b), Soy PC (c), DSPC (d), DODMA (e) with respective numeration used in NMR peak assignments

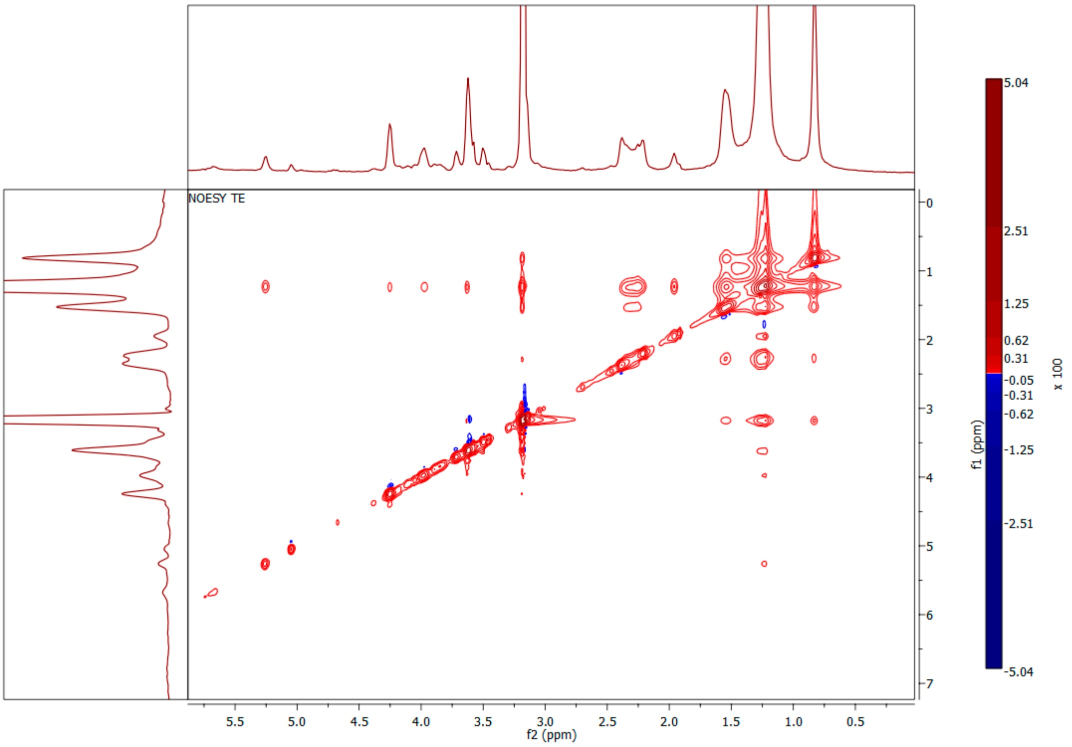

(a)

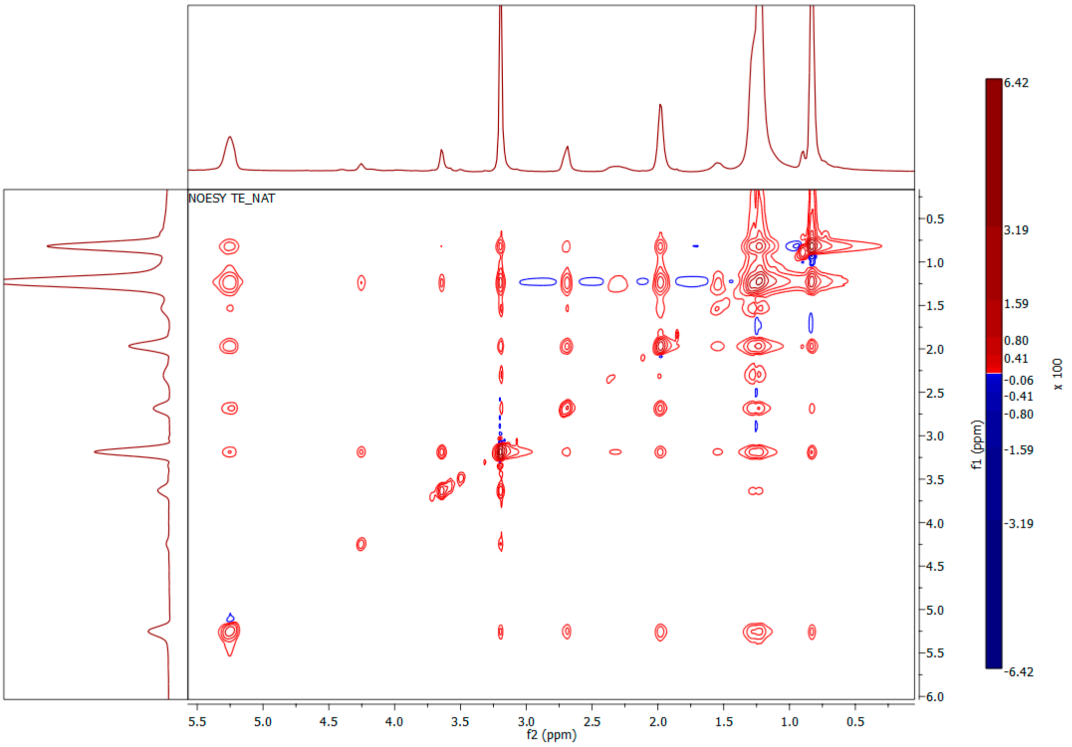

(b)

Figure S2: NOESY spectra of transethosomes (a) and Natamycin-loaded transethosomes (b).

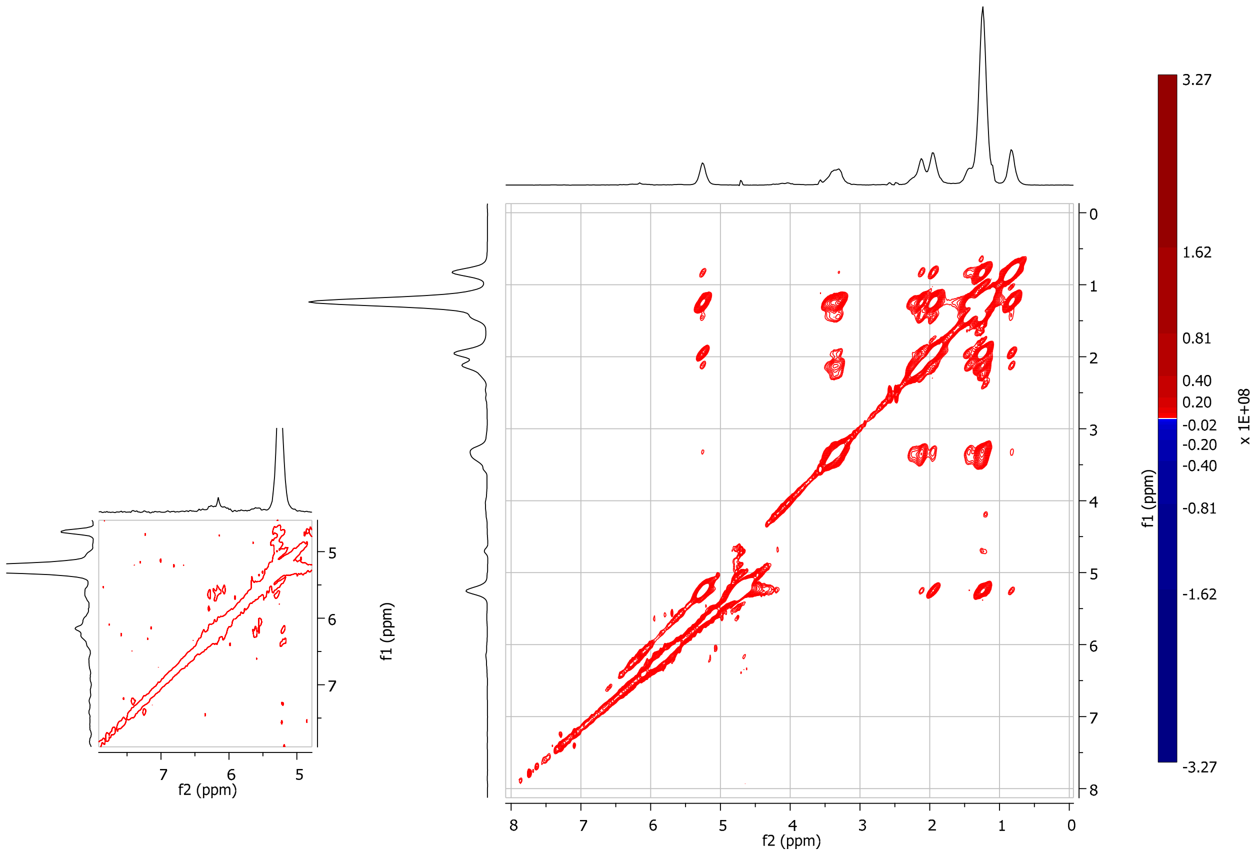

Figure S3: NOESY spectra of representative NAT-loaded LNP (right) with enlargement (left) of aromatic signals range.
